# Charitable giving and lay morality: understanding sympathy, moral evaluations and social positions

**DOI:** 10.1111/1467-954X.12332

**Published:** 2015-08-20

**Authors:** Balihar Sanghera

**Affiliations:** ^1^ University of Kent

**Keywords:** charitable giving, lay morality, sympathy, reflexivity, social inequalities

## Abstract

This paper examines how charitable giving offers an example of lay morality, reflecting people's capacity for fellow‐feeling, moral sentiments, personal reflexivity, ethical dispositions, moral norms and moral discourses. Lay morality refers to how people should treat others and be treated by them, matters that are important for their subjective and objective well‐being. It is a first person evaluative relation to the world (about things that matter to people). While the paper is sympathetic to the ‘moral boundaries’ approach, which seeks to address the neglect of moral evaluations in sociology, it reveals this approach to have some shortcomings. The paper argues that although morality is always mediated by cultural discourses and shaped by structural factors, it also has a universalizing character because people have fellow‐feelings, shared human conditions, and have reason to value.

This paper examines how charitable giving offers an example of lay morality, reflecting people's capacity for fellow‐feeling, moral sentiments, personal reflexivity, ethical dispositions, moral norms and moral discourses (Sayer, 2010). It will show that while moral evaluations can be sensitive to people's social positions and cultural scripts of what and who deserve attention, moral evaluations can also be *less* sensitive to the social positions of those making the evaluations and of those being evaluated. People can deliberate on the basis to be the person that they have reason to want to be. The paper will argue that the ‘moral boundaries’ framework underestimates the importance of fellow‐feelings (or sympathy), common human conditions and reason to value in shaping moral judgements.

Charitable giving is largely neglected in social science, partly because sociology tends to be cynical of people's normative practices, casting doubt on their genuine moral concerns, and trying to reveal the economic and political motives for their purportedly disinterested behaviour. For instance, Bourdieu's (1984, 1990) work on symbolic capital, habitus and social field offers a remarkable framework for analysing how social actors’ cultural practices can conceal forms of symbolic domination that help to reproduce social inequalities. But people's moral evaluations are missing in his work, which focuses instead on the mechanics of the deliberate mis‐recognition of the economic in cultural and aesthetic tastes (Lamont, 1992; Sayer, 2005). On the few occasions Bourdieu writes about philanthropy, he is cynical and dismissive (see also Silber, 2009; Graeber, 2011). Similarly, Foucault's (1980, 2006) work on discourses and power provides an impressive framework for understanding how people's subjectivities are socially constructed according to political rationalities and social arrangements, which are themselves forms of subjugation and control. Foucault himself was scathing of the mental health reforms pioneered by 19th century philanthropists, dismissing their humane actions as a way of controlling patients, replacing chains with compassion (see also Fraser, 1981).

While it is right that sociology exposes the different ways in which power and domination are manifested and misrecognized in social practices and relationships, there is a danger of overreach and committing disciplinary imperialism (Sayer, 2000). There are some good reasons to be wary of attempts to sociologize and reduce morality to social constructs and power relations. While moral concerns and values are always understood and articulated through existing cultural discourses, and are always conditioned by historical and structural factors, they are often about things of significance for human flourishing that exist independently of our conceptions of them. If this were not so, we could hardly make mistakes about moral evaluations, and revise them in light of personal experiences of disappointment, frustration and pain. Furthermore, if ethics is no more than a form of symbolic domination and power, on what basis can we adjudicate between competing ethical claims? Why would giving money to a children's charity be better or worse than contributing to a racist organization? By having a conception of human flourishing and harm, moral relativism and an ‘anything goes’ approach can be avoided (see Nussbaum, 2000; Sayer, 2003).

In this paper, I will examine how charitable giving offers an example of what Sayer (2011) terms lay morality, as understood by ordinary individuals and shaped by cultural and social factors. The first section will explore the nature of lay morality, offering some critical remarks on the concept of moral boundaries. I will describe the research design and methods in the second section. In the third section, key findings will be discussed, in particular examining how charitable giving reflects social actors’ different moral concerns and social circumstances. Finally, I will make some concluding remarks.

## Theoretical frameworks on charitable giving

This section will begin by examining how Lamont's (1992, 2000) theoretical framework on social and moral boundaries addresses charitable giving. After offering some criticisms of the concept of moral boundaries, it will suggest an alternative approach based on the idea of lay morality.

### Moral boundaries, volunteering and altruism

The recent theoretical development on boundary work by Lamont (1992, 2000) and Kefalas (2003) provides a useful understanding of how class, ‘race’ and the nation‐state can shape social and moral evaluations, generating a range of judgements on work, social stratification, neighbourhoods, the home, the polity, patriotism, volunteering and altruism. Social actors evaluate themselves in relation to others using socio‐economic, cultural and moral criteria, such as income, power, tastes, intelligence, manners and personal and religious values. They draw social, moral, cultural and racial boundaries that allow them to achieve a sense of dignity and moral worth by constructing similarities and differences between themselves and others. Broad cultural repertoires and historical and structural conditions help social actors to construct exclusionary boundaries between ‘us’ and ‘them’.

In her cross‐cultural studies on white and black working and upper‐middle‐class men in the US and France, Lamont (1992, 2000) describes how working‐class men feel economically inferior to upper‐middle‐class men, lacking money, status and influence, but consider themselves to be morally superior because they make sacrifices for the family ahead of personal achievements and value friendship over competition. Although white and black working‐class men agree on their criticisms of upper‐middle‐class men, who are characterized as being selfish, overly competitive and disingenuous, their evaluations of poor and disadvantaged groups differ. The African‐American history and experience of slavery, racial discrimination and the civil rights movement are reflected in black working‐class men's structural understanding of social inequality and poverty, in contrast to the individualist perspective offered by their white working‐class counterparts. Black men are sympathetic, caring and supportive towards poor and homeless groups, valuing solidarity and cooperation and not feeling morally superior to them. In contrast, Lamont argues, white American working‐class men draw upon a different set of cultural repertoires, including the fantasy of the American dream, moral individualism and the Protestant work ethic (cf. Graeber, 2007, 2011). In developing a strong sense of self‐responsibility and discipline, they criticize poor, disadvantaged and black groups for lacking such qualities, and accuse them of being irresponsible, dishonest, lazy and undeserving of welfare assistance (Lamont, 2000). For instance, white working‐class men describe the homeless as having ‘given up’, resent that poor groups receive help from the state, and associate black people with crime and welfare dependency.

Lamont (2000) finds that altruism in the US is not framed in terms of social solidarity and common humanity, but rather as being practical and technical, based on individualistic and religious beliefs. Volunteering and donations aim to provide immediate relief on a case‐by‐case basis, designed to alleviate the recipients’ pain and suffering, rather than to tackle underlying injustices. Lamont shows how having an individualistic understanding of inequality and poverty, white working‐class men distinguish between deserving and undeserving recipients of charity, associating the latter with immoral behaviour, such as drugs, alcohol and sex. She maintains that it is their religious faith and teachings, not principles of social justice and solidarity, which extol the moral virtues of compassion and generosity.

Whereas African‐Americans draw a weak moral boundary towards poor and marginalized groups that allows them to perform charitable works to assist them, white working men are individualistic and draw strong moral and social boundaries against people below them (Lamont, 2000). White working‐class men are ambivalent about charity, and are moved by local and personal motivations. Kefelas (2003) in her study on a white working‐class community in Chicago argues that volunteering and altruism (eg, faith groups and civic neighbourhood associations) are anchored to local issues (such as children's sport activities, security and public cleanliness) that help to ensure a moral order and stability in the community. White working‐class families have precarious economic lives, and feel threatened by the uncertainty and fear arising from their close proximity to poor and black neighbourhoods. Unable to escape to white middle‐class suburban areas, they are protective of their community, fearing that an encroachment of black and poor families into the area would have a detrimental impact on house prices and their retirement saving plans.

In contrast to white working‐class men, Lamont (1992) argues that white American upper‐middle‐class men view charities and voluntary associations as a way of gaining respect in the community, establishing their social reputation, and becoming part of the local elite. Beside the intrinsic satisfaction that charitable giving and volunteering can provide, they have an instrumental value, being an important source of friendship given the white elite's high degree of geographical mobility. Lamont (1999: 60) notes that charity can also have a reciprocal value, allowing the white upper‐middle‐class to ‘give back’ to the community, though the ‘community’ is equated with ‘people like us’, people defined by common ethnicity, religion and proximity. But, she argues, French white upper‐middle‐class men have a different perspective on charity, objecting to it for political reasons: poor and disadvantaged groups have a right to be helped, and should not be forced to rely on the whims of the rich or individual paternalistic sentiments. They think that the state has a collective responsibility to support marginalized groups. For these reasons, Lamont argues that French white upper‐middle‐class men do not regard volunteering as a good indicator of moral character but, instead, they express their compassion through their willingness to support a strong welfare state.

Although the boundary work framework offers some insights into how class and ‘race’ can shape charitable giving and volunteering, it does have some limitations. First, whereas cultural and socio‐economic positional goods (such as tastes, education, income and power) tend to be monopolized by particular social groups operating in competitive social spheres with the aim to exclude or dominate others, morality is not a positional good. It is odd to think that just because a social group has more of a particular moral virtue, say compassion, others are likely to have or enjoy it less. Furthermore, morality has a universalizing character, in that social actors usually argue that their moral evaluations, reasons and virtues are good not only for them, but also for others, irrespective of class, gender and ‘race’. Unlike cultural and aesthetic judgements, moral evaluations matter because they are about how to treat and be treated by others and affect people's subjective and objective well‐being. People undertake actions and activities to be the person that they have reason to want to be. In contrast to the cynical depiction of American people as being morally individualistic and self‐interested, Graeber (2007, 2011) argues that the American society is better conceived as a battle over access to the means to behave altruistically. Lacking cultural and social capital to pursue higher, more noble, career goals, say, in the art world, the third sector, education, politics or journalism, many white and black American working‐class people join the army, believing that it will offer them an opportunity to live a life of adventure and camaraderie, in which they will do something genuinely noble and do good in the world (eg, giving free dental examinations to villagers).

Second, the metaphor of moral boundaries ignores the capacity of social actors to have sympathy for others, to imagine their situations, and to approve or disapprove of their actions (Smith, 1976). Without sympathy, social interaction would be difficult, and moral sentiments cannot emerge. Sympathy also enables social actors to criticize members of their own class and racial community for immoral behaviour (eg, dishonesty and child abuse), and to praise the moral virtues and actions of those from other communities. Making judgements of what is good and bad, just and unjust need not be constrained by class or racial boundaries.

### Lay morality

Sayer (2005) notes that lay morality refers to how people should treat others and be treated by them, matters that are important for their subjective and objective well‐being. While power, interests, reciprocity, discourses, discipline, socialization, habits and conventions are useful for an external description of people's actions, they miss the first person evaluative relation to the world and the force of their evaluations (Sayer, 2011). Lay morality reflects ‘hard’ and ‘soft’ ethical issues of justice and rights and of care and love. It implies an everyday, rather than a philosophical, conception of the good life, which is always understood and articulated through existing cultural resources and discourses, and is shaped by historical and structural conditions. While lay morality can be formalized in social norms and rules and discursively articulated and legitimatized, it also takes on a visceral and embodied form of moral sentiments and dispositions, learnt and developed through ongoing social interaction, as described by Smith (1976). Some scholars, such as Bourdieu (1984) and Lamont (1992, 2000), describe how social and moral evaluations are sensitive to and influenced by social status, but Sayer (2005) argues that moral judgements can also be *less* sensitive to people's social positions. As already noted, moral evaluations can have a universalizing character.

Lay morality is a product of several interacting elements, which are socially variable (Sayer, 2010). First, moral sentiments (including compassion, self‐interest and indignation) are fallible but intelligent responses to situations that affect individuals’ moral concerns and goals, such as familial relations, practical achievements and social values (Archer, 2000). Sentiments are not merely subjective, but are about things that have real impact on human flourishing and social suffering (Nussbaum, 2001). Immoral sentiments (such as contempt, arrogance and submissiveness) also occur, often arising from situations of power asymmetry. Moral and immoral sentiments possess causal powers, moving and motivating people to take actions in response to their situation. As people's emotions reflect their social experiences, differences in emotions emerge. For instance, class inequalities tend to produce negative sentiments, such as working‐class shame and envy, and middle‐class contempt and anxiety. Ray (2014) shows that shame is situated within structural and cultural conditions that are likely to evoke shame, and that class shame can move humiliated and despised working‐class men to riot and loot, both performing an act of defiance against the legitimate culture and expressing normative justifications for transgression.

Second, as Sayer (2010) argues, fellow‐feeling (or the ability to understand others) is a pre‐condition of everyday moral conduct, and is crucial for social interaction. Through experiencing or imagining certain good and bad events themselves, people have a degree of insight into others’ experiences and situations, enabling them to compare and evaluate their own and others’ actions. The capacity for fellow‐feeling is variable with some groups, such as autistic individuals, finding sympathy and social interaction difficult. Moreover, sympathy can be countered by moral blasé; for example, contemporary societies are saturated with media reports and images of suffering and tragedy that can result in emotional and political indifference to others’ appalling conditions (Boltanski, 1999).

Third, social actors acquire particular moral dispositions (or virtues and vices), which are learnt and repeated behaviour (Sayer, 2010). For instance, trainee teachers and nurses learn to be attentive to the needs of their pupils and patients, developing over time caring dispositions. Early socialization, moral education and social context can also shape people's moral traits; for example, strong child–parent attachment and humanist teaching can encourage children to be trusting, honest, generous and respectful towards strangers. Class, gender and ‘race’ are also likely to affect people's moral dispositions and behaviour; for example, a middle‐class sense of entitlement and superiority, gendered care and modesty, and racial distrust and loyalty (see also Reay, 2006; Gilligan, 1990; Lamont, 2000).

Fourth, norms are an important guide for what conduct is and is not appropriate without the need for too much deliberation (Smith, 1976). Moral norms are distinguished from other norms because they have significant implications for human flourishing (Sayer, 2010). For instance, religious almsgiving, such as Christian tithes and Islamic ‘zakat’, is not merely a religious observance for believers, but is also about something independent of itself, namely, how people can live together in which they have regard for each other's well‐being (see also Mahmood, 2003). Moral norms are always contestable, as different groups struggle over ideas of what constitutes human well‐being.

Fifth, morality is always culturally mediated: moral discourses, stories, myths, fantasies and symbols provide people with ways to understand, shape and reason about how to treat and be treated by others (Sayer, 2011). Moral discourses are always contested, reflecting diverse, complex and often conflicting ideas about human dignity and flourishing. Social actors can be mistaken in thinking that particular discourses have good argumentations for achieving human well‐being, which exists independently of their particular conceptions of it. The relationship between moral discourses and social inequalities is also complex, sometimes resulting in harm and suffering. For instance, Skeggs (1997) argues that while working‐class women demand to be treated with equal respect and dignity, they develop their own moral form of respectability, but lacking the necessary cultural and economic capital, they sometimes experience fear, humiliation, resentment and hatred.

Sixth, individuals are reflexive beings, who interpret the world in relation to things that matter to them, deliberating and prioritizing multiple moral concerns and commitments (Archer, 2007). Reflexivity ranges from intense, and sometimes critical, deliberations through to more fleeting and fragmented reflections as part of the stream of consciousness. The more important the decision, the more intense the deliberation, as social actors are likely to ask themselves how effective their actions will be and how others will view them (Sayer, 2010). People are attentive to how they and the things they care about are faring and how others regard them. Furthermore, personal reflexivity is socially variable; for example, individuals who have a stronger commitment to social justice than to, say, practical achievements are likely to engage in more critical internal conversations on the mis‐match between their life projects and goals and their social milieu (Archer, 2007).

It is worth noting that the lay morality approach avoids two common mistakes in conceptualizing moral evaluations. First, it rejects a strong version of social constructionism, which reduces morality to cultural discourses and power, and ignores the importance of the social ontology of a human being as a vulnerable, interdependent, needy and evaluative person (Sayer, 2004). Strong social constructionism is unable to make sense of how social actors can be mistaken about what constitutes human well‐being, and their attempts to revise their ideas and conduct in light of their subjective and objective ill‐being. Second, it avoids a strong version of human essentialism, which posits that human qualities are fixed and universal, and fails to consider how ongoing social interaction and inequalities can shape moral evaluations. Morality is always interpreted through available cultural resources, and human qualities – such as moral sentiments and dispositions – are socially variable and contingent. But while moral judgements can be sensitive to social positions, they can also be insensitive to them, having a universalizing character.

## Research design and methods

Between 2008 and 2009, I conducted in‐depth interviews with 41 individuals from a range of occupations, including public sector administrators, university lecturers, social care workers, home‐keepers, self‐employed workers, mature students and retirees. Most of the interviews were conducted in Kent, south‐east of the UK. Almost all the interviewees were recruited through mass emails to several local public and charity organizations, asking people to participate in a research project on giving and volunteering. All those who responded were subsequently interviewed. The sample was not completely random, as five interviewees were specifically approached in order to have more ‘black’ and middle‐class donors in the sample.

The semi‐structured interviews consisted of two parts, lasting on average two and a quarter hours. The first part asked the interviewees to recount their life history, describing their life from their early upbringing and schooling through to their current family and work situation, their personal goals and their routines. Interview questions included, ‘Can you please tell me something about yourself from childhood and schooling to adult life’, ‘What were the important events or who were key people in your life?’, ‘Can you please describe your typical day and week’, and ‘Typically over a month or a year, can you say what do you do together with your neighbours / people on your street / members in the local community. Or maybe what you do *for* them?’ This part of the interview aimed to grasp how the interviewees understood and interpreted their own life; more specifically, what were their key moral concerns, what were they attentive to, how did these things change over time, and how were charitable acts embedded into their daily, weekly or monthly routines.

In the second part, interviewees were asked to recall their acts of giving and volunteering, and to describe their feelings and motivations. Interview questions included, ‘Recall an incident when you gave money or time to a charity, a cause or someone to help out, talk me through how it began, what were you thinking and feeling?’, ‘What were the reasons and motivations for this particular action and its timing?’, ‘Can you say something about whether your friends, family members and work colleagues give money or volunteer?’, ‘Of the money and time you have given to things, causes or people, which one has meant the most and the least to you?’, and ‘Can you say why giving or helping out matters to you?’ This part of the interview aimed to grasp how charitable acts are understood and interpreted, what reasons and motivations were given for them, and how other people shaped their donations. Overall, a picture emerged of how interviewees navigated their way through the world, being attentive to things of importance to their well‐being, and to what extent charitable giving was a meaningful and significant activity in their lives.

The interviewees were assigned a social class using multi‐dimensional criteria: social class upbringing (working or middle‐class parents based on their occupations), educational qualifications (school, college or university), occupation (unskilled, semi‐skilled, skilled or professional), and economic household situation (struggling to make ends meet, managing to cope or having a comfortable lifestyle). In addition, some interviewees defined themselves into a particular social class, sometimes using euphemisms such as ‘common as muck’, ‘being privileged’ and ‘don't worry about money’. Based on the interview data, the sample consisted of 19 working‐class people, 16 lower‐middle‐class and 6 upper‐middle‐class. Working‐class participants possessed a much lower volume of cultural, social and economic capital than their middle‐class counterparts, and the upper‐middle‐class participants had significantly greater cultural and economic capital (in particular education and profession) than the lower‐middle‐class participants. [Correction added on 15 April 2016, after first online publication: Sample data figures which were previously wrongly stated as 21, 13 and 7 have now been corrected as 19, 16 and 6 respectively in this version.]

In terms of gender composition, 25 women, 15 men and one transgender person participated in the research. The sample also consisted of five ‘black’ interviewees (three British‐Asians, one African immigrant and one Iranian immigrant) and three ‘white’ immigrants (one Argentinean, one Greek Cypriot and one South African). The rest were white British, of whom one was Welsh. The study also had seven retirees, of whom one was a part‐time lay clergy and three were involved in managing local civic associations (a table tennis club, a residents’ housing association and a naval heritage charity). There were two young undergraduates, just about to complete their degrees. Most interviewees were young and middle‐aged adults, and a few were approaching retirement.

All interviews were digitally recorded, and interviewees were reassured about confidentiality and anonymity. The interviews were transcribed, and then the transcripts were returned to them to check and edit. Only a few made slight alterations to the text, correcting minor factual details. The subsequent analysis was based on themes I developed after reading the transcripts a couple of times. Most themes, such as ‘giving money’, ‘giving time’, ‘values’, ‘faith’, ‘tithes’, ‘caring for others’, ‘character’, ‘reflections upon giving’, ‘informal giving’ and ‘why giving matters’, were shared across the transcripts. Some themes, including ‘justice’, ‘activism’, ‘strategic giving’, ‘self‐interest’, ‘family and children’ and ‘sympathy’, were more evident in some transcripts than others. After the data had been thematized, I was able to see similarities and differences among the participants, especially noting how significant and meaningful charitable giving was in their lives. For some, charitable giving played an important part in their lives, whereas for others it was incidental.

## The moral and social aspects of charitable giving

This section will discuss how the various elements of lay morality shape charitable giving, beginning with people's capacity for fellow‐feeling. Social actors can sympathize with others who have suffered injustice, having experienced similar or related forms of injustice (Sayer, 2010). Eve, a working‐class part‐time hospital porter, gives to Shelter, a homeless charity, because the issue is close to her heart, having been homeless herself and knowing homeless people to be vulnerable to events outside their control:

I feel passionate about homeless, it's because of stories like that and also people living in temporary housing and things like this, that's what really, really gets to me…. There was a period when my mum kicked me out, I was homeless and there's been periods where my sisters have been kicked out,… so it is kind of close to me…. For example, a single mother with three children whose other half batters her. She will have to go and get temporary housing to be housed away from him and then she'll be left in there for two year. That's not her fault…. That's just a bad situation, bad circumstances, bad luck really and a lot of the people who are homeless, it's just bad luck that's befallen them and so they need help really and the government doesn't help them and they fall through the net, so and it's sad and not enough people really care about it as far as I can see. There's too much of this, kind of, it's their own fault sort of idea.

In addition to being sympathetic and compassionate, Eve is angry that the government does not provide sufficient temporary and emergency accommodation. She is also critical of the way the media often blame homeless people for their own plight without understanding how bad luck affects their lives, some coming from broken and abusive homes. Her donation to Shelter reflects both compassion and justice. She is attentive to homelessness in several other ways. For instance, she supports her local church's initiative to provide temporary accommodation to homeless people, gives cigarettes and sits next to people sleeping rough outside, and sometimes invites some of them to her home to sleep on the sofa.

Women's experience of abuse and violence can heighten their sensitivity to such and related forms of suffering and injustice, moving and motivating them to take actions (Walker, 2006). Four female interviewees describe how as children they had been physically or sexually abused for several years, leaving them traumatized, resentful and distrustful, unable to speak to others about their experience until they were much older. Rachel, a working‐class house‐keeper, who had been sexually abused as a child, regularly donates to the National Society for the Prevention of Cruelty to Children (NSPCC), hoping that the charity will protect children from harm and will enable abused children to talk to someone to get help:

I do like you know, give money to NSPCC that's just, I have done that for years…. I think that they do a great job…. It's just part of my life, that's what I do… I kind of think there are children out there that are being abused and everything and they need, it's a really good charity that help people. There is that part of me that thinks that you know I never come across when I was, when I needed it, but you would hope, I don't know that, you know that they do, that they are trying to protect children aren't they?

Giving to NSPCC is part of Rachel's life because of her childhood experience. She understands what abused children go through, the sense of loneliness, uncertainty and fear. She had wished that there had been more support for her, rather than feeling abandoned by people around her. She criticizes her school for not delving further into her truancy and poor academic performance, noting that she had been an A‐grade pupil before the abuse began. Rachel is also a school governor at her children's school and fundraises for their local majorettes club, so that they and other children can travel abroad to perform. While her charitable activities benefit her children, she also feels an obligation towards other children:

I do lots of things in school like the reading and everything, the teachers haven't got time to read to the children anymore and the two classes I go to it's nothing to do with my children. I could say I only want to do my own children, but you know to be honest I like reading to some of the others, getting to know some of the other children and helping them. So, yeah I think it's good in that way I can go into school, I haven't got it that everything's got to be around my own, I quite like that.

Rachel is sympathetic towards other children, who are in the similar situation to her own children. Her sense of care expands beyond her own family circle towards others in the local school. As Smith (1976) notes, sympathy and a feeling of moral responsibility are easier to elicit the closer others’ situations are to one's own.

While relations of care have many positive qualities, they are also associated with power and strongly gendered, with women undertaking much of the burden of care (Tronto, 1994). Women's sympathy for the plight of others can produce feelings of guilt and responsibility, resulting in them accepting care responsibilities for fear of not letting others down (Skeggs, 1997). Madeleine, a middle‐class estate agent, felt pressurized into becoming a supervisor of a local Scout group because it did not have anyone managing it and was threatened with closure. She felt obligated to help local mothers who struggle with the daily pressures of family and work life and who were dependent upon her to keep the Scout group running:

A lot of mothers always feel guilty that you're not doing enough for your children…. I was depressed for a number of years and probably wasn't the best mother in the world, and I'm thinking maybe I can make up the shortfalls that I had with my children with other children, and that will somehow compensate…. I sort of think maybe there are other people going through those kinds of hard times and I would like somebody to be able to do that for my children, to be able to give them a good experience when I'm having a hard time. So if I can do it for other people's children, then maybe because we have got children that come that obviously don't always have the happiest of times so just for that one hour I can be super‐mum or super‐leader [at the Scout group].

Madeleine reflects on how she and other parents are vulnerable and needy beings, and are dependent upon each other. Her maternal guilt moves her to become a ‘super‐mum’, providing relief and help to other parents at the Scout club. While such sentiments and roles are clearly gendered and socially constructed, they also reflect an internal normative force relating to human fragility, neediness and interdependency. Charitable giving, and more generally lay morality, fuses ethical concerns with cultural scripts and social positions. As Sayer (2011) notes, morality and power can be discussed simultaneously, without reducing the former to the latter. This understanding of charitable giving, and more generally moral evaluations, is quite different to Lamont's moral boundaries approach, which also understates the significance of gender.

Of course, recognition of injustice and suffering can come from political discourse, as well as from personal experience. People are reflexive about what values should guide them (Archer, 2000). Kamela, an upper‐middle‐class information technology manager, is an active member of her local community. She regularly donates money to her parish church, volunteers as a member of the Independent Monitoring Board which inspects prison conditions, and helps with fundraising activities at her local school and church fetes. She notes that people depend upon various associations and communities to flourish, and condemns individualism for impoverishing society:

Because no man's an island. You know we're all interconnected, and most of us wanna go through life with people being nice to us, and not being sort of cynical and thinking ‘Oh, I'm alright Jack!’… We're not gonna have the best society we can, unless people are prepared to give…. We went a lot wrong in our society in the Thatcher years, when everybody thought it was okay to make lots of money and ‘I'm alright Jack, and stuff you!’, and what does it matter if I make a fortune on the back of three million unemployed, you know, I'm okay, my family's okay. You know, it really annoys me when people say charity begins at home, what you mean just within that four walls, you know, what is home? Home's big? You know, I believe in communities, I believe in church communities, school communities, you know, guides and cubs and that sort of thing…. So I just think we're a poorer society if we don't give.

Kamela's charitable activities are framed in terms of communal and collective values, critical of social and moral ideas that fail to appreciate how people are interconnected and rely on each other for care and support. She believes that an excessive focus on the self and the family can threaten social solidarity and civil society, and laments how people's lives are often sacrificed for profit.

Sayer (2010) argues that the capacity for fellow‐feeling means that even though social actors may appear to be self‐absorbed for much of the time, they usually tend to help, respect and be friendly to others some of the time. Moral stories, religious celebrations and national events (such as biblical parables, Christmas and Armistice Day) can alter people's consciousness in the direction of unselfishness, connecting consciousness to moral virtue (see also Murdoch, 1970). Paul, a former working‐class commercial engineer now retired, donates to several Christmas charities:

I'd also give, you know, to certain Christmas charities because I think, you know, there's always a tradition in Britain that at Christmas you like to think of people who are not as fortunate as you. And when I was a child, when we had our Christmas dinner, my parents, you know, would always think of people who were less fortunate than ourselves, because I think it's very important that you do that, you know, at least I think if you're thinking about people it's better than not thinking about them…. Some people would probably say, ‘Well you can afford to give a lot more than that!’ Well maybe I could, but I give… I'm doing something and because it's a part of the way I was brought up, what Christmas is about, you know and the way we are.

Paul was brought up to reflect upon Christmas as an important time of year to think about less fortunate groups, evoking acts of generosity. In part, the Nativity story reminds people to show kindness to strangers, the homeless and those in distress. The media sometimes emphasize the moral significance of Christmas, reporting on how local religious communities and charities organize soup kitchens and temporary accommodations for the homeless.

Smith (1976) argues that given that human beings are prone to self‐love and self‐deceit, moral norms and rules provide an important source of guidance on how to act in particular situations. For instance, several interviewees try to adhere to religious norms on almsgiving, which stipulate that a percentage of their income must be donated to their place of worship for community service and public benefit. Often the rules are not strictly followed, causing individuals to feel guilty, as Ravinder, a British‐Asian lower‐middle‐class police clerk, explains:

I give money to the temple I go to, because in my faith, I'm supposed to give one tenth of my earning to God in appreciation of what God has given me to thank him, to say thank you for your blessings, for your mercy or your kindness, whose creation is yours for which we are thankful. It's like saying grace at a table, at the meal, saying thank you Lord for your food or whatever, and I'll give money once a year to the temple and the temple uses the money to various projects that they have to help the community both here and in India…. I don't do one tenth, and I feel guilty for not doing one tenth, but it's a question of having to afford one tenth, as to can I afford to give one tenth, and I feel I can't afford to give one tenth, so I give what I can afford.

Ravinder believes that she has a religious duty to give one tenth of income to her local Sikh temple in gratitude to God's blessings and mercy, but she is unable to fulfil it completely. Instead she gives as much as she can. Giving cannot be read off from religious norms, as individuals always reflect upon them, having a degree of personal autonomy and power in deciding whether to follow, repudiate or adapt them (Archer, 2000). It is worth noting that Ravinder's duty to give is not only an act of religious devotion, but also, as she notes, a way for the temple to support vulnerable and needy members of the local Sikh community and poor groups in India, reflecting people's moral obligations to disadvantaged and marginalized groups.

Lay ethical practices are not merely the product of moral sentiments, concerns, norms and reflections, but are also unevenly structured according to people's relative status, power and interests in the social field (Sayer, 2011). Sensitivity about social recognition and symbolic power can be reflected in their charitable giving, in that rich and middle‐class people are more likely to engage in particular forms of giving and volunteering that validate and legitimize their social position (Curtis, 1997; Shapely, 2001). The study shows that middle‐class people tend to over‐value charitable causes and practices (such as education, the arts, international development and professional skills) associated with the dominant symbolic culture, and to under‐value things (including charity shops, crafts and manual labour) associated with the subordinate culture. Zoë, an upper‐middle‐class university lecturer, enjoys giving occasional lectures for free to the Workers’ Educational Association, a non‐profit adult educational institute:

I tend to do some teaching for the Workers’ Educational Association…. I'll teach for nothing…. It's just a really good thing to be doing, everyone's getting a lot out of it, people like it. I get a lot out of it too…. It's great to be able to see people engaging and taking off.

While teaching English literature *pro bono* is partly motivated by democratic and progressive ideals to educate mature adults who otherwise would not have access to formal education, Zoë’s actions also serve to valorize her cultural capital, receiving praise and social recognition from others. Rather than opting for cynical or reductionist interpretations of giving as disguised self‐interested or class behaviour, it is more accurate to explain it as motivated by mixed sentiments (namely, sympathy, justice and self‐interest) and multiple concerns, including practical achievement and social causes, recognizing that people are sometimes disposed to over‐value things that are agreeable and advantageous to their situation (Smith, 1976).

People's moral expectations of themselves and others partly depend upon how they attribute responsibility and blame for social issues and circumstances. Sayer (2011) argues that in determining responsibility people often underestimate the role of social influences on what they and others are able and motivated to do. Several white interviewees believe that poverty and underdevelopment in low‐income countries are due to corrupt and wasteful actions of governments and international agencies, underestimating the effects of global finance, trade flows, corporate power and geo‐politics on national economies. Richard, a former lower‐middle‐class publicity officer now retired, does not donate to international development charities, being very critical of aid programmes in Africa:

As for these charities that are installing pumps in African villages, Africa must be knee deep in pumps by now, for 50 years people have been contributing their ten bob or whatever it was to put a pump in an African village, there can't be that many villages, so somebody somewhere is doing very nicely out of this and it's probably the pump manufacturer…. We were giving independence to countries that were nowhere near ready for independence, all that happened was that they became over‐ruled by despots and none of them were as well off as they were when they were part of the British Empire and certainly Zimbabwe is a classic example of this, if you wanted an excuse for empire that would be it.

Richard sneers at the idea that international development charities can produce good work, feeling that the charity industry and African state officials are corrupt. He ridicules many African countries, believing that they were not ready for independence and would have been better served if they had remained within the British Commonwealth. His account depoliticizes economic development by minimizing the effects of post‐colonial and global economic factors, and highlighting instead the failings of African leaders and state officials. While attributing responsibility and blame can be a product of moral reasoning, it can also be structured according to people's relative positions in the social field, so that social class, gender and ‘race’ have a bearing upon how they think about social issues and problems.

## Conclusion

I have examined how lay morality can provide some insights into the nature of charitable giving, reflecting people's capacity for fellow‐feeling, moral sentiments, personal reflexivity, ethical dispositions, moral norms and moral discourses. The paper aimed to avoid strong forms of social constructionism and essentialism: the former overlooks the importance of the social ontology of human beings as vulnerable, independent and needy individuals, who interpret the world in relation to their moral concerns; and the latter ignores how social actors always describe their situations through available cultural resources, and are shaped by historical and structural factors. The paper also argued that the concept of moral boundaries fails to appreciate how people's moral evaluations are good not only for them, but also for others, irrespective of class, gender and ‘race’, largely because of sympathy and the universalizing quality of morality. This does not mean that people's moral judgements are not socially variable; as we demonstrated, charitable giving is inflected by class, gender, ‘race’ and ‘ethnicity’. Nor does it mean that their moral evaluations are without problems; as we saw, social inequalities and prejudices can be reflected in charitable giving, helping to reproduce structural injustices.

Some readers may be reluctant to embrace ideas of common human conditions (or the social ontology of human being) and morality's universalizing quality to cut across social positions, pointing out that moral evaluations are socially constructed and variable across history and cultures, and that power and interests shape morality. Sayer (2011) notes that this reluctance can arise from the fear that acknowledging such things implies essentialism, psychologism, determinism or homogenization, though, as we have noted, social variability is quite compatible with the social ontology of human being and transnational values. Another reason for the reluctance can be driven by sociological imperialism, meaning that sociology aims to extend its reach to the exclusion of other disciplines, imagining that it is the most fundamental and insightful social science (Sayer, 2000).

The irony is that there are always implicit moral evaluations in sociological descriptions and explanations of social reality, usually in the form of condemnations of suffering, domination, violence and harm inflicted upon sentient and social beings. Such ethical evaluations matter, otherwise why would sociologists care to examine the effects of social structures and inequalities. Moreover, people, including sociologists, criticize others for mis‐treating them, and justify to others the things that matter to them, arguing that respect, dignity, fairness, justice and equality have a degree of validity, objectivity and truth beyond their own social group. Of course, their articulations and evaluations of what constitute human well‐being are contestable and fallible, suggesting that it exists independently of their particular conceptions of it, otherwise there would be nothing to discuss and debate about. While moral judgements and practices are inflected by power and interests, they are not determined by them, otherwise adjudicating between competing sets of moral values would become either arbitrary or an exercise of power.

Finally, let me address another source of unease that some readers may have. The paper implies that social actors can be mistaken about their moral evaluations of people and things, over‐valuing and belittling objects that help to validate and legitimize their social position and symbolic dominance in the social field. Readers may ask on what basis, if at all, good or bad judgements can be made. Sociologists often explicitly avoid both judging the subjects they study and proposing ideas for a flourishing or damaging life, possibly for fear of being labelled unscientific, ethnocentric or essentialist. Though they invariably slip normative values into their analysis, recognizing particular forms of power to be oppressive and harmful – this partly explains their motivation to study issues like domestic abuse and racism. Without knowing what and why particular things are damaging or valuable, sociologists’ critique of power and domination loses meaning (Sayer, 2003). While sociologists may be reticent about making value judgements, they are usually much clearer in their own personal lives. More research is required into how lay ethical practices are often confusing and contradictory, partly because ordinary individuals think and act in piecemeal fashion, so that their actions tend to be inconsistent with their beliefs and values.

Please quote the article DOI when citing SR content, including monographs. Article DOIs and “How to Cite” information can be found alongside the online version of each article within Wiley Online Library. All articles published within the SR (including monograph content) are included within the ISI Journal Citation Reports® Social Science Citation Index.
